# Synergistical Use of Electrostatic and Hydrophobic Interactions for the Synthesis of a New Class of Multifunctional Nanohybrids: Plasmonic Magneto-Liposomes

**DOI:** 10.3390/nano9111623

**Published:** 2019-11-15

**Authors:** Gabriela Fabiola Știufiuc, Ștefan Nițică, Valentin Toma, Cristian Iacoviță, Dietrich Zahn, Romulus Tetean, Emil Burzo, Constantin Mihai Lucaciu, Rareș Ionuț Știufiuc

**Affiliations:** 1 Faculty of Physics, “Babeș-Bolyai” University, M. Kogălniceanu 1, 400084 Cluj-Napoca, Romania; gabi.stiufiuc@phys.ubbcluj.ro (G.F.Ș.); romulus.tetean@phys.ubbcluj.ro (R.T.); emil.burzo@phys.ubbcluj.ro (E.B.); 2 MedFuture Research Center for Advance Medicine, “Iuliu Hațieganu” University of Medicine and Pharmacy, L. Pasteur 4-6, 400349 Cluj-Napoca, Romania; stefan_nitica@yahoo.com (Ș.N.); valentin.toma@umfcluj.ro (V.T.); 3 Faculty of Pharmacy, “Iuliu Hațieganu” University of Medicine and Pharmacy, L. Pasteur 4-6, 400349 Cluj-Napoca, Romania; cristian.iacovita@umfcluj.ro (C.I.); clucaciu@umfcluj.ro (C.M.L.); 4 Semiconductor Physics, Chemnitz University of Technology, D-09107 Chemnitz, Germany; zahn@physik.tu-chemnitz.de

**Keywords:** multifunctional nanohybrids, magneto-liposomes, superparamagnetic nanoparticles, gold nanoparticles, hyperthermia

## Abstract

By carefully controlling the electrostatic interactions between cationic liposomes, which already incorporate magnetic nanoparticles in the bilayers, and anionic gold nanoparticles, a new class of versatile multifunctional nanohybrids (plasmonic magneto-liposomes) that could have a major impact in drug delivery and controlled release applications has been synthesized. The experimental results confirmed the successful synthesis of hydrophobic superparamagnetic iron oxide nanoparticles (SPIONs) and polyethylene glycol functionalized (PEGylated) gold nanoparticles (AuNPs). The SPIONs were incorporated in the liposomal lipidic bilayers, thus promoting the formation of cationic magnetoliposomes. Different concentrations of SPIONs were loaded in the membrane. The cationic magnetoliposomes were decorated with anionic PEGylated gold nanoparticles using electrostatic interactions. The successful incorporation of SPIONs together with the modifications they generate in the bilayer were analyzed using Raman spectroscopy. The plasmonic properties of the multifunctional nanohybrids were investigated using UV-Vis absorption and (surface-enhanced) Raman spectroscopy. Their hyperthermic properties were recorded at different frequencies and magnetic field intensities. After the synthesis, the nanosystems were extensively characterized in order to properly evaluate their potential use in drug delivery applications and controlled release as a result of the interaction with an external stimulus, such as an NIR laser or alternating magnetic field.

## 1. Introduction

The use of different types of multifunctional nanoparticles (NPs) for biomedical applications, a field known as nanomedicine, is in the limelight of current research activities. Its principal aim is to use nanometric structures and devices for diagnostic and therapeutic purposes at a molecular level [1,2]. As oncologic diseases are amongst the principal causes of worldwide mortality, and given the fact that their conventional therapy is accompanied by serious adverse reactions, it is unanimously accepted that new therapeutic alternatives, mainly based on multifunctional nanohybrids, hold promise toward solving a major part of these issues.

The most promising nanomedicine applications in cancer therapy are represented by the detection, targeting, and ablation of malignant tissues through minimally invasive methods based on specific nanohybrid interactions using an external stimulus, such as a magnetic field and/or NIR radiation [1,3]. In principle, these objectives are feasible if one considers the unique properties possessed by different types of nano-objects proposed so far for such applications and the specific features of the tumoral tissue. Amongst them, its increased capacity for retaining objects smaller than 500 nm as a result of its leaky blood vessels with large fenestrations (a phenomenon known as the enhanced permeation and retention effect) and its increased sensibility to elevated temperatures compared to healthy tissues are the more important ones [2,3,4,5,6]. So far, several classes of nano-objects have emerged as promising candidates for cancer therapy. Plasmonic nanoparticles, magnetic nanoparticles, and liposomes have been intensively studied, and some products based on these nanoparticles have already been approved for clinical use [7,8]. The most important factors that still need to be addressed and understood for their large-scale use in clinical practice are the development of new synthesis strategies that are able to generate complex nanohybrids in a very reproducible fashion, as well as a rigorous physico-chemical characterization of these nanohybrids.

Plasmonic nanoparticles possess free surface electrons that give rise to collective oscillations upon interaction with electromagnetic radiation of a certain wavelength [9,10,11,12,13]. This phenomenon, known as localized surface plasmon resonance, depends on the characteristics of the nanoparticles (such as size, shape, and surface features) [14,15], and gives rise to unique properties (electromagnetic field amplification, photothermal effect) that opens the possibility for numerous applications in the theranostic field [16]. Gold nanoparticles are perhaps the most-studied class of plasmonic nanoparticles. Due to their increased stability and the possibility of plasmonic properties and surface characteristics tuning, the major envisaged applications for them are in biomedical imaging and detection [17], targeted drug delivery [18,19,20], gene expression regulation [18], and photothermal therapy [6].

Magnetic nanoparticles, especially those made from iron oxides (maghemite—γFe_2_O_3_, and magnetite—Fe_3_O_4_), present good chemical stability, biocompatibility, and hyperthermia properties when exposed to an alternating magnetic field. Superparamagnetic nanoparticles are preferred for biomedical applications as a result of their capacity to generate heat when exposed to magnetic fields and for their ability to completely reduce their magnetization after exposure to external magnetic fields [21]. Their direct applications are mainly found in tumor ablation through magnetic hyperthermia and contrast enhancement in magnetic resonance imaging [22]. A specific application is derived from their property to accumulate at sites where an external magnetic field is applied. In this way, not only the magnetic nanoparticles, but also the structures that are attached to their surfaces tend to accumulate at those sites. The possibility to incorporate these nanoobjects in liposomes is a major advantage as they can be directed to the desired target and the encapsulated substances can be released at that site under the influence of an external magnetic field [21].

Liposomes are small vesicles with an aqueous core that are comprised of one or more lipidic bilayers organized in such a manner that their hydrophilic regions come in contact with water whereas the hydrophobic portions come in contact with each other [4,23,24,25]. They possess biocompatible membranes with features similar to the cellular ones, lacking antigenic and pyrogenic properties [4,26]. Due to their structure, liposomes can encapsulate both hydrophilic and hydrophobic molecules, protecting them both at the same time from environmental degrading factors, such as enzymes. Therefore, liposomes can be employed as targeted transport systems for various therapeutic substances, and are amongst the first nanosystems approved for clinical use [3,8].

The major drawback for nanoobjects in in vivo applications is the interaction with the immune system and/or blood proteins. One simple method of avoiding and delaying their clearance is PEGylation (i.e., attaching polyethylene glycol molecules to their surface). Polyethylene glycol (PEG) is a nontoxic hydrophilic polymer lacking immunogenicity. It forms a steric barrier on the surface of the nanoobjects that prevents their interaction with opsonins and other blood proteins (such as the complement). In this way, PEGylated nano-objects possess a stealth character and are able to freely circulate in the bloodstream for longer periods of time [4,27,28,29,30].

Even though they possess promising characteristics, nano-objects have several further specific drawbacks that limit their practical applications. A strategy to overcome these issues is found in combining the features of different classes of nano-objects by synthesizing hybrid structures that comprise their functionalities. Over time, our research group has developed several original strategies for the synthesis of biocompatible nanoparticles exhibiting very interesting surface properties by using PEG [31,32,33,34], natural cyclodextrins [35], and plant extracts [36] as reducing agents. Meanwhile, by taking advantage of electrostatic interactions, an original method for the synthesis of PEGylated plasmonic liposomes was reported [37].

The aim of this study is to take advantage of the previous achievements and to develop an original strategy for the synthesis of a new class of hybrid nanostructures, namely plasmonic magneto-liposomes, by decorating cationic liposomes incorporating hydrophobic superparamagnetic iron oxide nanoparticles (SPIONs) in the bilayer with spherical hydrophilic gold nanoparticles. After the successful synthesis of multifunctional nanohybrids, they are characterized by means of absorption spectroscopy, magnetic measurements, transmission electron microscopy, photon correlation spectroscopy, nanoparticle tracking analysis, and (surface-enhanced) Raman spectroscopy. By carefully analyzing the Raman spectra acquired on the magneto-liposomes containing different amounts of SPIONs, it has become possible to confirm the presence of these nanoparticles inside the bilayers, together with the modifications they induce at the nanoscale level. To the best of our knowledge, this paper reports the first successful synthesis and characterization of such a complex multifunctional nanohybrid.

## 2. Materials and Methods

### 2.1. Chemicals

Tetrachloroauric (III) acid, iron (III) acetylacetonate, oleic acid, oleylamine, 1,2-hexadecanediol, tetramethylammonium hydroxide solution 25 wt.% in H_2_O, chloroform, and benzyl ether were purchased from Sigma-Aldrich (Merck Group, Darmstadt, Germany). Polyethylene glycol 1000, trisodium citrate dihydrate, and methylene blue were obtained from Carl Roth (Karlsruhe, Germany). 1,2-Dioleoyloxi-3-trimethylammonium-propane chloride and soybean phosphatidyl-choline were purchased from Lipoid GmbH (Ludwigshafen, Germany). Sodium hydroxide micropearls were provided by Lach-Ner and double-distilled water (18.2 MΩ) was used as a solvent.

### 2.2. Preparation of Superparamagnetic Iron Oxide Nanoparticles

The SPIONs synthesis was performed by adding 1 mmol of iron(III) acetylacetonate, 6 mmol of oleic acid, 6 mmol of oleylamine, and 2 mmol of hexadecanediol in 20 mL of benzyl ether. The mixture was stirred for 30 min, then placed in a stainless-steel container. After nitrogen flushing, the container was sealed and placed in a furnace. The mixture was gradually heated (3 °C/min) to 200 °C, maintained at this temperature for 120 min, then heated to 300 °C with the same temperature gradient. After 90 min, the container was removed from the furnace and the mixture was left to cool at room temperature (RT).

Given the fact that magnetic hyperthermia measurements were performed in aqueous media and the obtained nanoparticles were hydrophobic, a hydrophilization step was required prior to the hyperthermia measurements. As such, the obtained hexane-dispersed SPIONs were precipitated with ethanol and separated using a neodymium magnet. The residual ethanol was removed via rotary evaporation. The obtained powder was ultrasonicated in a 25% (*w*/*w*) tetramethylammonium hydroxide aqueous solution and incubated overnight. The NPs were magnetically separated, and the process was repeated several times. In the final steps, the SPIONs were resuspended in double-distilled water and used for magnetic hyperthermia investigations.

### 2.3. Liposomal Dispersion Synthesis

The synthesis of cationic liposomes was performed using a thin-layer evaporation method of a chloroform solution containing dioleoyloxi-3-trimethylammonium-propane chloride (DOTAP) and soybean phosphatidyl-choline (SPC) in a 2:1 molar ratio. Briefly, 14.2 mg of DOTAP and 34.8 mg of SPC were dissolved in 30 mL of chloroform and introduced into a rotary evaporation system in order to completely remove the solvent. The obtained lipidic film was hydrated by the addition of double-distilled water, followed by a gentle hand-shaking of the mixture for 5 min. The resulting opalescent aqueous dispersion was further ultrasonicated for 30 min. At the end of the process, the dispersion became transparent, a sign that indicates the formation of small liposomes.

Magneto-liposomes were synthesized using the same procedure, by adding different volumes (10, 50, 250, and 1000 μL) of hexane dispersion of superparamagnetic nanoparticles in the chloroform lipid solution.

### 2.4. PEGylated Gold Colloid Synthesis 

Gold colloid synthesis was performed by heating an aqueous mixture of tetrachloroauric (III) acid and a reducing agent (PEG 1000). Briefly, 1 g of PEG 1000 and 1 mL of NaOH 1% solution were added to 48 mL of double-distilled water. The obtained solution was heated in an Erlenmeyer glass to 85 °C, then 0.55 mL of a 128 mM H[AuCl_4_] aqueous solution was added under vigorous stirring. The mixture was further heated to the boiling temperature, then 0.5 mL of NaOH 1% solution was added. The color of the mixture rapidly changed from pale yellow to ruby red, indicating the successful formation of the gold colloid.

### 2.5. Plasmonic Liposomes Synthesis 

The decoration of the liposomes with gold nanoparticles was performed by mixing 990 μL of the gold colloid with 10 μL of liposomal dispersion. The rapid color change from red to violet indicated the successful attachment of the gold nanoparticles to the liposomal surface.

### 2.6. Methods

The nanoparticles, liposomes, and nanoparticles-liposomes complexes were characterized using UV-Vis absorption spectroscopy, transmission electron miscroscope imaging, X-ray diffraction, photon correlation spectroscopy (PCS), zeta-potential measurements, (surface-enhanced) Raman spectroscopy, magnetic measurements, and magnetic and plasmonic hyperthermia measurements.

#### 2.6.1. UV-Vis Absorption Measurements

The Vis absorption spectra were recorded using a T92+UV–VIS Spectrophotometer from PG Instruments (Lutterworth, UK). The absorption curves were acquired on standard plastic cells at room temperature, over a spectral range between 300 nm and 800 nm. The spectral resolution was set at 2 nm.

#### 2.6.2. Transmission Electron Microscopy (TEM) and Energy Dispersive Spectroscopy (EDS) Measurements

Electron microscopy measurements were performed on a HT7700 (Hitachi, Tokyo, Japan) Transmission Electron Microscope operating at 100 kV, using the high-resolution operation mode (spot size 3 = 0.60 μm). Samples were deposited on carbon films on top of copper grids for 2–5 min depending on the sample type and concentration. After deposition, the excess solution was blotted away using No. 42 ashless filter paper and the grids were left covered at room temperature to completely dry. A negative contrast was not used as the SPIONs provided sufficient contrast for imaging. The obtained images were calibrated for size, annotated, and processed (contrast enhancement using histogram stretching, smoothing (sigma = 2.2), and cropping) in ImageJ free software (U.S. National Institute of Health, Bethesda, MD, USA).

#### 2.6.3. X-ray Diffraction Measurements

X-ray diffraction (XRD) measurements were carried on powder samples at room temperature using a Brucker D8 Advance diffractometer (Bruker, Billerica, MA, USA). The Cu Kα radiation was employed for all measurements. The lattice parameters were calculated using FullProf software (FullProf Suite, Institute Laue-Langevin, Grenoble, France).

#### 2.6.4. Photon Correlation Spectroscopy Measurements

The photon correlation spectroscopy (PCS) analysis was performed on a Vasco^γ^ nanoparticle analyzer (Cordouan Technologies, Bordeaux, France) using a monochromatic laser beam with a wavelength of 658 nm and 65 mW power. Zeta potential measurements were taken using a Wallis^ζ^ Zeta potential analyzer (Cordouan Technologies, Bordeaux, France).

#### 2.6.5. (Surface-Enhanced) Raman Spectroscopy (SERS) Measurements

The Raman measurements were recorded using a confocal Renishaw^®^*inVia* microscope (Renishaw plc, Wotton-under-Edge, UK), equipped with a Leica microscope (Leica Microsystems GmbH, Wetzlar, Germany), using a 100× objective (N.A. 0.85). A 785 nm diode laser (Renishaw, Wotton-under-Edge, UK) has been used for excitation. Prior to each set of measurements, a calibration procedure was performed using an internal silicon reference. The laser power (measured at the substrate surface) was ≈65 mW and the acquisition time was set between 3 and 10 s. The spectrograph was equipped with a 1200 lines/mm grating and a charge coupled device camera (CCD, Renishaw, Wotton-under-Edge, UK). The spectral resolution of the spectrometer was 0.5 cm^−1^.

The SERS measurements performed on liquid solutions were recorded in 1 mL glass vials filled with 540 µL of colloid and 60 µL of analyte (methylene blue solutions of multiple concentrations) in the 200–2000 cm^−1^ range, using a DeltaNu Advantage spectrometer (DeltaNu, Laramie, WY, USA) equipped with a laser diode emitting at 785 nm. The laser power was 100 mW and the spectral resolution was 5 cm^−1^. Each SERS spectrum was the average of 10 recordings taken with an acquisition time of 10 s.

#### 2.6.6. Magnetic Measurements

Magnetic measurements were performed in the 2–300 K temperature interval in an external magnetic field of up to 2 T using a vibrating sample magnetometer (Cryogenic LTD, London, UK). Magnetic hyperthermia measurements were recorded using an Easy Heat System (Ambrell, New York, NY, USA). Alternating magnetic fields with strengths up to 65 kA/m and frequencies between 100 and 400 kHz were employed.

## 3. Results and Discussions

The strategy presented here for the creation of multifunctional plasmonic magneto-liposomes is a two-step process consisting first in the synthesis of unilamellar cationic magneto-liposomes as a result of the hydrophobic interactions between SPIONs and a liposomal lipidic bilayer. Subsequently, they were decorated using PEGylated plasmonic nanoparticles based on electrostatic interactions between the previously synthesized cationic magneto-liposomes and the anionic PEGylated gold nanoparticles. The synthesis and characterization of the two major building blocks used for the creation of multifunctional nanohybrids (hydrophobic SPIONs having a diameter less than 12 nm and spherical PEGylated gold nanoparticles) is described in the following sections.

### 3.1. SPIONs

**Figure 1:**
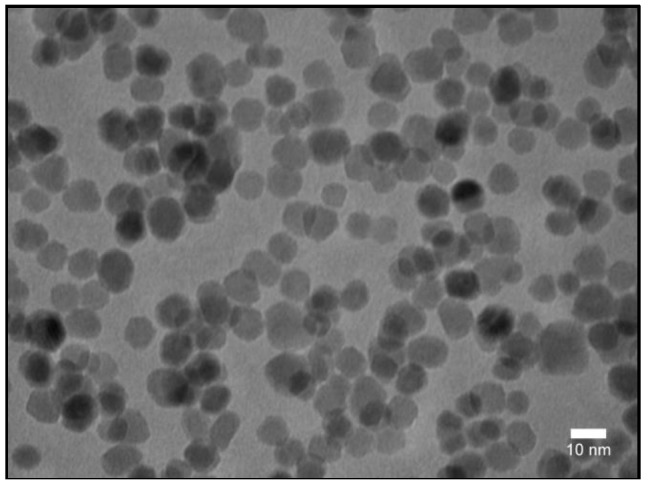
TEM image of the as-synthesized Fe_3_O_4_ SPIONs.

Spherical Fe_3_O_4_ magnetic nanoparticles were synthesized using a “classical” thermal decomposition method, as described in the experimental section. The as-synthesized hydrophobic spherical Fe_3_O_4_ SPIONs were highly monodisperse, as shown in the TEM image of Figure 1. A statistical analysis performed on the TEM images indicated a mean diameter of ≈12 nm. In order to investigate their crystalline structure, X-ray diffraction measurements were performed on powder samples. The XRD pattern (Figure S1) clearly reveals the existence of a pure inverse spinel crystalline structure.

The position and the relative intensities of all diffraction peaks can be ascribed to magnetite Fe_3_O_4_ [38]. No FeO or Fe_2_O_3_ peaks were found in the XRD pattern, indicating that all magnetic nanoparticles (MNPs) consisted of pure magnetite Fe_3_O_4_. The black color of the powder was a further confirmation that the samples were of a pure magnetite phase. The calculated lattice parameter a = 8.373 Å is very close to that of bulk magnetite (a = 8.375 Å) [31].

The crystalline size of the Fe_3_O_4_ nanoparticles, calculated from the (311) diffraction peak using Debye–Scherer’s formula, is 13.2 nm, which is close to the average diameter obtained from the TEM images.

The hyperthermia properties of the magnetic nanoparticles are based on the variation of the specific absorption rate (SAR) value with the alternating magnetic field amplitude and frequency. Therefore, a high SAR value indicates good hyperthermia properties. The obtained SPIONs exhibited a maximum SAR value of 550 W/g (for a magnetic field amplitude of 65 kA/m at a frequency of 380 kHz), which is in good agreement with the data reported in the literature for spherical magnetite nanoparticles of a comparable size in similar alternating magnetic field conditions [39,40,41].

**Figure 2:**
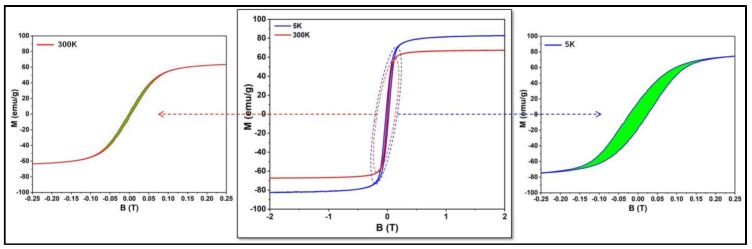
Hysteresis curves recorded at 5 and 300 K for the SPIONs samples.

The magnetic hysteresis measurements presented in Figure 2 demonstrates that the magnetic nanoparticles were ferromagnetic at 5 K and superparamagnetic at room temperature (300 K). At low temperatures (5 K), the SPIONs showed a hysteresis curve that is typical for a pure ferromagnet. At this temperature, the saturation magnetization (M_s_) had a value of 80 emu/g that decreased to 60 emu/g at RT. The M_s_ decrease with increasing temperature is explained by an increase in thermal vibrations (and thus randomization of the aligned magnetic moments) and is described by Bloch’s equation. Small deviations from the Bloch behavior were most probably due to spin canting effects that were very pronounced in the case of small spherical MNPs. The coercive field measured at 5 K had a value of 200 mT (left inset in Figure 2). It decreased close to 0 T at RT, indicating that at this temperature, the Fe_3_O_4_ MNPs may be considered to be in a superparamagnetic state (right inset in Figure 2). This behavior can be also seen in the zero-field-cooled (ZFC)/field-cooled magnetization curves that started to join at around 100 K, well below RT. The maximum in the ZFC curve, which corresponds to the average onset of the ferromagnetic to superparamagnetic transition (the so-called blocking temperature T_B_), was located at 59 K (Figure S2).

### 3.2. PEGylated Gold Nanoparticles

**Figure 3:**
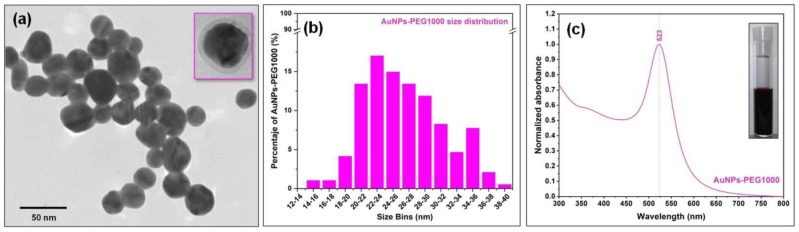
Transmission electron microscopy (TEM) image of PEGylated gold nanoparticles (**a**); TEM image of a single gold nanoparticle showing the presence of the polyethylene glycol (PEG) layer surrounding the nanoparticle (NP) (inset a). Statistical distribution of NPs sizes calculated from TEM images (**b**). UV-Vis absorption spectrum of the gold colloid (**c**); optical image of the gold colloid (inset c).

Anionic PEGylated gold nanoparticles were synthesized using an original method developed in our laboratory, employing PEG 1000 molecules as both reducing and capping agents [34]. The synthesis method is described in the experimental section. The as-obtained PEGylated gold colloids had a spherical shape and exhibited a localized surface plasmon resonance peak at 524 nm, as can be seen in the absorption spectrum presented in Figure 3.

It is known from literature that the diameter and concentration of the colloidal solutions containing spherical gold nanoparticles can be theoretically estimated from their absorbance values measured at the (localized surface plasmon resonance) LSPR frequency (A_lspr_) and at 450 nm (A_450_), respectively [42]. The values calculated using this theoretical model for nanoparticles diameter and concentration were ≈25 nm for the NP diameter and 2.5 × 10^12^ NPs/mL in the colloidal solution. These theoretical values were in very good agreement with the statistical analysis performed using the TEM images (Figure 3) and with the experimental data provided by the nanoparticle tracking analysis (NTA) (data not shown). The plasmonic properties of the PEGylated AuNPs were previously tested by acquiring surface-enhanced Raman (SER) spectra of methylene blue (MB) in aqueous solutions, in a 10^−3^–10^−7^ M concentration range. The limit of MB detection in these experimental conditions, using spherical PEGylated AuNPs as SERS substrates, was 10^−6^ M [34].

Moreover, the presence of PEG molecules on the outer surface of the gold nanoparticles was proven by means of FT-IR spectroscopy [34]. Briefly, FT-IR spectra acquired on a dry film of purified gold colloid presented the same vibrational bands as pure PEG 1000. In addition, a vibrational peak at 1656 cm^−1^, corresponding to an asymmetric stretch mode of an unidentate gold-carboxylate bond, was identified in the spectrum indicating that the PEG molecules suffered an oxidative transformation during the reduction process of the gold(III) ions [34].

### 3.3. Magneto-Liposomes

The second step in the synthesis of multifunctional magneto-plasmonic nanohybrids consisted in the creation of cationic magneto-liposomes by incorporating SPIONs into the liposomal lipidic bilayer. The two classes of lipids employed in this study for the synthesis of cationic liposomes were a cationic one (DOTAP) and a neutral one (SPC) that were mixed in a 1:2 molar ratio. These two lipids have similar structures, as both present a quaternary ammonium cation, two ester groups, and two fatty acid chain residues. Besides the similarities, each compound has characteristic features. DOTAP has two identical 18 C monounsaturated fatty chain residues (oleoyl chains) while SPC presents one 16 C saturated fatty chain residue (palmitoyl chain) and one 18 C double unsaturated fatty chain residue (linoleyl chain). Another difference between these two compounds is the linkage between the quaternary ammonium group and the fatty acid chain residues. While in the DOTAP structure, this linkage is provided by a propane 1,2-diol residue, in the SPC structure, a negative phosphoethanol residue intercalates between the quaternary ammonium group and the propane 1,2-diol residue, which explains the fact that the SPC molecule has an overall neutral charge, in contrast to the positively charged DOTAP molecule (Figure S3).

**Figure 4:**
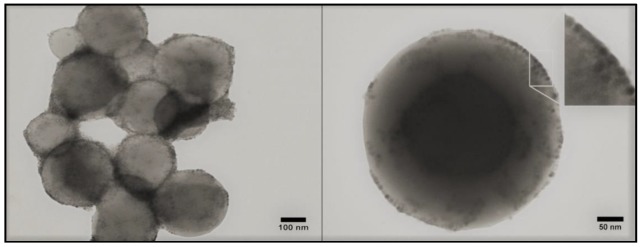
TEM images of magneto-liposomes (MLP50) (**left**); unstained TEM image of a single magneto-liposome (MLP50) (**right**). The inset magnifies a membrane portion highlighting the incorporation of SPIONs in the lipid bilayer.

The hydrophobic SPIONs were incorporated in the lipid bilayer in the course of the thin lipid film formation during a rotary evaporation process. Following hydration and ultrasonication procedures, cationic nanoliposomes containing SPIONs in their bilayer were obtained. In order to evaluate the SPION interaction with the lipidic bilayer, several SPION concentrations were tested. The yellow-brownish color of the dispersions increased with increasing quantities of SPIONs (Figure S4). This observation can be considered as a first proof of the successful incorporation of MNPs in the liposomal lipidic bilayer, as those nanoparticles cannot be homogenously dispersed in aqueous media due to their hydrophobic nature. Typical TEM images of the magneto-liposomes are presented in Figure 4. The stability of the gold colloid and liposomal dispersions were evaluated using zeta potential measurements (Table S1). As expected, gold nanoparticles possessed a negative zeta potential, whereas the bare liposomes had a positive one. If one considers a stability threshold of ±25 mV [37], it can be stated that all colloidal dispersions were physically stable.

The experimental values provide further evidence for the occurrence of electrostatic attraction forces between the two classes of nanoobjects, which will be further employed for the decoration of liposomes using plasmonic NPs. The hydrodynamic sizes of the liposomes were evaluated by means of PCS measurements (Table S2). The PCS spectra of every batch of liposomes exhibited two peaks: a very intense one located above 150 nm and a small one located below 100 nm. These peaks were first attributed to the presence of two populations of liposomes: a major one having hydrodynamic diameters above 150 nm and a small population of lipidic vesicles having a mean diameter of ≈30 nm. The insertion of small quantities of SPIONs in the liposomal membrane did not affect their dimensions, as the pure liposomes had similar dimensions as those from the 10 μL batch. The increase of SPION quantity led to a size increase of the large liposomes until a saturation point was reached. Consequently, the large liposomes from the 250 μL batch exhibited hydrodynamic diameters similar to those from the 1000 μL batch (≈390 nm). Another interesting observation was the increasing size of small vesicles starting from the 250 μL batch. These phenomena may suggest that the SPIONs were preferentially located in the large liposomes’ membrane, as its curvature was smaller and the membrane tension was weaker. When the SPIONs quantity increased, the size of the large liposomes also increased until they reached a saturation point. In our experiments, this point could be identified by interpolation between the 50 μL and 250 μL batches, as the size of the large liposomes remained practically unaltered for the 1000 μL batch. Given the fact that the sizes of large liposomes suffered a greater increase with increasing SPION quantities before the saturation point compared to the size of small liposomes after the saturation point, one can presume that small liposomes existed only before the saturation point was reached. After that, the excess nanoparticles organized as clusters stabilized by a micellar layer of lipids.

Raman spectroscopy was employed as a further proof of the SPIONs’ incorporation into the lipidic bilayer, as well as for giving new insights concerning the interaction of SPIONs with the fatty acid tails, which were the main constituents of the lipidic bilayer. Therefore, the Raman spectra recorded on pure (unloaded) DOTAP/SPC liposomes were compared with those acquired on DOTAP/SPC liposomes incorporating different amounts of SPIONs in their lipidic bilayer: 10, 50, 250, and 1000 µL.

**Figure 5:**
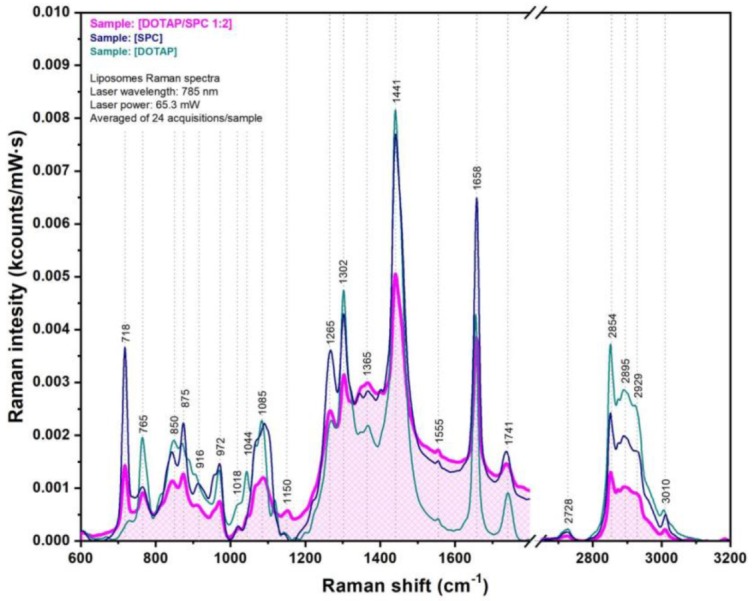
Raman spectra of dioleoyloxi-3-trimethylammonium-propane chloride (DOTAP) lipids (green spectrum), soybean phosphatidyl-choline (SPC) lipids (blue spectrum), and DOTAP/SPC liposomes (magenta spectrum).

The Raman spectra of the two classes of lipids (SPC and DOTAP) employed for the synthesis of the cationic liposomes, together with the spectrum of pure 1:2 DOTAP/SPC liposomes, recorded on dry samples using a 785 nm excitation laser, are presented in Figure 5. As seen in Figure 5, the Raman spectrum of the liposomes contained all the vibrational peaks characteristic for the two classes of lipids used in the synthesis process. This is clear evidence of the presence of these lipids in the liposomal membrane. The only major difference between the two lipids was the 718 cm^−1^ peak, assigned to the symmetric stretch vibration of the C–N bonds from the N^+^(CH_3_)_3_ choline group, which can be found only in the case of phosphatidylcholine (SPC). In the high wavenumber spectral region (2700–3100 cm^−1^), characteristic for carbon chains vibrations, five major peaks (2728, 2854, 2895, 2929, and 3010 cm^−1^) and a shoulder (2960 cm^−1^) were present. According to References [43,44], the 2728 cm^−1^ peak can be assigned to in-plane scissoring deformation vibrations and out-of-plane wagging deformation vibrations of the of CH_2_ group, and the 2854 and 2895 cm^−1^ peaks can be attributed to symmetric and antisymmetric stretching vibrations of the same group. The 2929 cm^−1^ peak and the 2960 cm^−1^ shoulder can be assigned to symmetric and antisymmetric stretching vibrations of CH_3_ groups. The 3010 cm^−1^ peak can be found only in the case of unsaturated fatty acids and can be assigned to =C–H stretching vibrations. From the Raman spectrum of unloaded liposomes, one can deduce that the vibrations of the CH_2_ groups dominated the spectrum, probably due to their higher density in the lipidic bilayer. In the fingerprint region, 700–1750 cm^−1^, the Raman spectrum of pure liposomes can be divided in two regions. The first region (700–900 cm^−1^) was dominated by the presence of four major peaks: 718, 765, 850, and 875 cm^−1^. According to Czamara et al. [43], the 718 and 875 cm^−1^ peaks can be assigned to choline group, while the 765 and 850 cm^−1^ bands can be attributed to phosphate group vibrations. It must be noted that both these functional groups can be found on the exterior surface of the liposomes. The positive surface charge, responsible for the cationic character of the liposomes, is also “located” on these exterior groups, making them the first moieties that will interact with anionic PEGylated AuNPs during the electrostatic-driven decoration step. The second spectral region (900–1750 cm^−1^) was dominated by several pronounced vibrational peaks (972, 1265, 1302, 1444, 1658, and 1741 cm^−1^) and a broader band around 1085 cm^−1^. These were all assigned to vibrations of CH, CH_2_, and CH_3_ groups, as well as C=C (1658 cm^−1^) and C=O (1741 cm^−1^) bond vibrations. The two double bonds and the majority of the above-mentioned groups were located inside the lipidic bilayer and could be affected by the presence of MNPs inside the bilayers. The complete assignment of the major vibrational bands recorded in the case of DOTAP/SPC cationic liposomes is presented in Table S3.

**Figure 6:**
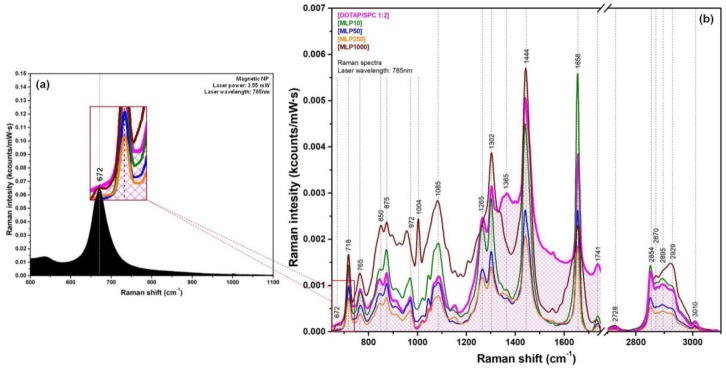
(**a**) The black Raman spectrum was recorded of the SPION powder. (**b**) Superposition of the Raman spectra recorded of pure (unloaded) liposomes (magenta curve) with those recorded of the four classes of MLPs.

The successful synthesis of the four classes of magneto-liposomes (MLPs) was driven by hydrophobic interactions between MNPs and the lipidic fatty tails upon addition of different volumes of MNPs in the lipid solution, according to the procedure described in the experimental section. Depending on the number of MNPs present in the lipidic bilayer, the as-synthesized MLPs will were denoted MLP10 (synthesized using 10 µL of MNPs), MLP50 (synthesized using 50 µL of MNPs), MLP250 (synthesized using 250 µL of MNPs), and MLP1000 (synthesized using 1000 µL of MNPs). The Raman spectra of all classes of MLPs are shown in Figure 6b, together with the spectrum of pure liposomes. The spectra were recorded on dry samples using a 785 nm laser. For a proper understanding of the origin of the small peak located at 672 cm^−1^ and observed only in the Raman spectrum of the MLPs containing the largest amount of MNPs (MPL1000), the Raman spectrum of pure MNPs is also presented in Figure 6a.

The liposomal Raman spectra are presented for the 650–3100 cm^−1^ spectral region and are compared with the Raman spectrum of pure (unloaded) liposomes recorded under the same conditions.

The incorporation of small amounts of MNPs into the bilayer seemed to have no marked influence on the Raman spectrum of MLP10 and MLP50 samples. Moreover, in the case of MLP10 samples, the inclusion of small amounts of MNPs inside the bilayer seemed to reinforce the liposomal structure, leading to a strong increase of the Raman signal recorded for these liposomes. Only for this class of MLPs was a strong intensity increase observed for the vibrational peak, which was assigned to C=C bond stretching vibrations (1658 cm^−1^). This double bond is characteristic for unsaturated fatty acids being located inside the membranes. It can thus be considered as a very precise indicator of the molecular interactions inside the bilayer and was previously used, together with the 1444 cm^−1^ peak, for assessing the structure of different lipidic compounds containing unsaturated fatty acids in their structure [43]. A similar behavior was observed in the case of MLP50 samples, suggesting that up to this concentration of SPIONs in the lipidic bilayer, the structure of MLPs remained unmodified. This observation was also confirmed by PCS measurements (Table S2). The two liposomal populations present in the PCS data had almost the same diameters in the case of unloaded MLPs (181 nm and 31 nm) and MLPs loaded with 10 µL (180 nm and 36 nm) and 50 µL (227 nm and 34 nm) SPION solutions.

By further increasing the number of SPIONs accommodated in the bilayers (MLP250 samples), no major differences were observed in the Raman spectrum. According to the PCS results, showing a two-fold increase in the diameters of the two liposomal populations in the case of MLP250 with respect to bare liposomes, one can conclude that the diameter of the major liposomal population increased with the number of SPIONs accommodated in the bilayers. Moreover, when the maximum quantity of SPIONs that could be accommodated in the bilayers was reached, the MLPs diameter also reached a saturation point. From the PCS data recorded in the case of MLP1000 samples indicating the presence of a dominant liposomal population having a mean diameter of 389 nm, one can conclude that under the used experimental conditions, the saturation diameter of the MLPs was ≈390 nm. This conclusion was supported by the values of the zeta potential measurements obtained for bare liposomes and for the four classes of MLPs (Table S1). The unloaded liposomes had a zeta potential value of +60 mV, whereas the two classes of MLPs (MLP10 and MLP50) that had undergone a self-organization process upon the introduction of small amounts of SPIONs into the membrane had very similar values, namely +43.9 mV and +43.4 mV, respectively. A further increase in the number of SPIONs led to a slight increase in the MLPs’ diameter and zeta potential values.

The last class of MLPs (MLP1000) contained the biggest number of SPIONs in the lipidic membrane. The incorporation of the MNPs in the membrane was proven by means of optical imaging, PCS measurements, and TEM, but the most spectacular result came from Raman spectroscopy. As seen in the Raman data in Figure 6b, at this concentration, the structure of the lipidic membrane was strongly affected by the large number of MNPs inserted into the bilayers. The strongest difference could be detected for the 1658 cm^−1^ peak (C = C stretching vibration), which became very broad, and for 1285 cm^−1^ (deformation vibration of the unsaturated =CH group), which transformed into a shoulder. In the high wavenumber region, the 2929 cm^−1^ peak (symmetric stretching vibration of the CH_3_ groups) became the dominant one, probably as a consequence of the fact that the vast majority of these groups were not located inside the bilayer. The vibrational bands affected by the presence of MNPs were assigned to bonds or groups located inside the bilayer. The four vibrational peaks assigned to choline and phosphate groups (718, 765, 844, and 875 cm^−1^), located on the outer surface of the MLPs, seemed to be unaffected or affected very little, even at this huge concentration of MNPs.

The most interesting effect, however, was the occurrence of the small 672 cm^−1^ peak, which started to be faintly visible in the case of MLP250, but became a distinct peak only in the case of MLP1000 samples. In order to understand the origin of this peak, Raman measurements were performed on pure SPION samples. A typical Raman spectrum of the SPIONs is presented in Figure 6a. As can be seen, the spectrum was dominated by one peak located at 672 cm^−1^, being characteristic of the pure magnetite Fe_3_O_4_ phase [45,46]. Therefore, we can conclude that the small 672 cm^−1^ peak could be attributed to the Fe_3_O_4_ MNPs present in large quantity in the bilayer of MLP1000 samples. Very surprisingly, these nanoparticles could provide a detectable Raman signal, even when they were embedded in the lipidic membrane (inset Figure 6a).

### 3.4. Multifunctional Plasmonic Magneto-Liposomes

**Figure 7:**
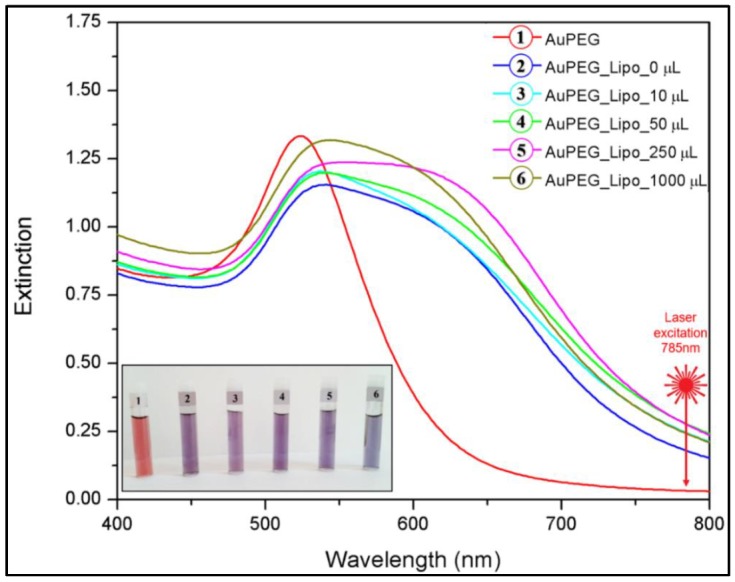
VIS absorption spectra of the PEGylated gold colloids (1), and of the complexes they formed with pure liposomes (2) and MLPs containing different amounts of MNPs in their bilayers (3–6). The inset shows the optical images of the gold colloids and their liposomal complexes with their respective liposomal batch number: 1–0 μL, 2–10 μL, 3–50 μL, 4–250 μL, 5–1000 μL. The samples were diluted four times before image recording.

The last step in the synthesis procedure of multifunctional magneto-plasmonic nanohybrids is the electrostatic-driven attachment of the PEGylated AuNPs on the surface of the magneto-liposomes. As can be observed in the optical image presented in the inset of Figure 7, after mixing the gold colloids (vial 1) with unloaded cationic liposomes (vial 2) and with the four classes of magneto-liposomal dispersion (vials 3–6) previously synthesized and characterized, their color shifted from red to purple-blue. Bathochromic shifts emerged in the absorption spectra in visible range of the liposomal complexes, which confirmed the visual observations (Figure 7). This phenomenon indicated the attachment of gold nanoparticles on the liposomal outer surface, followed by a coupling of their surface plasmons. The instantaneous color change observed in all cases was a clear sign of a successful attachment of gold nanoparticles to the liposomal surface.

**Figure 8:**
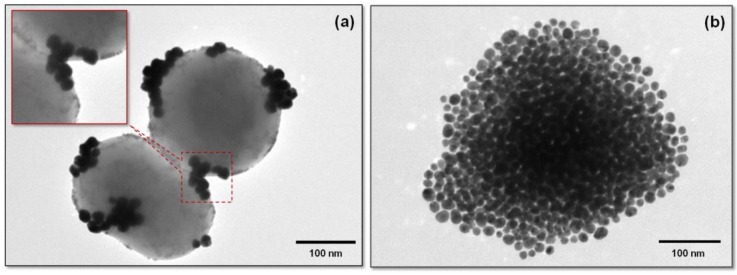
TEM image of incomplete decorated magneto-liposomes (MLP50). The inset highlights the presence of PEGylated AuNPs on the outer surface of the liposomes and of SPIONs inside the lipidic bilayer (**a**). TEM image of a single plasmonic magneto-liposome (MLP50) (**b**).

A second confirmation of the successful decoration process came from TEM measurements. As can be seen in the case of a single completely decorated MLP (Figure 8b), the PEGylated AuNPs were uniformly distributed on the outer surface of the MLPs. This complete coverage screened the detection of the small SPIONs located inside the bilayer. The situation was completely different when TEM experiments were performed on partially decorated MLPs. A typical image of such partially decorated MLPs is presented in Figure 8a. PEGylated AuNPs, which tended to organize in small plasmonic clusters, were present on the external surface of the liposomes, as also observed in the case of fully decorated Multifunctional Magneto Plasmonic Nano Hybrids (MMPNHs).

For a proper understanding of the collective plasmonic properties of newly synthesized multifunctional nanohybrids, photothermal measurements were performed on PEGylated gold colloids and fully decorated MMPNHs using a 785 nm excitation laser. The experimental procedure was similar to that employed for the calculation of magnetic SAR values of SPIONs, but in this case, the external excitation employed for heat generation was a 785 nm laser instead of an alternating magnetic field. The photothermal data obtained in the case of PEGylated AuNPs and fully decorated liposomes are shown in Table S4.

As can be predicted from the Vis absorption spectra presented in Figure 7, PEGylated AuNPs showed an almost 50% increase of their SAR values (from 320 W/g to 450 W/g) when attached to the liposomal surface. This is strong evidence of the occurrence of collective plasmonic modes as a consequence of a strong interparticle coupling mediated by their individual electrostatic interactions with the liposomes.

Very interestingly, the incomplete decoration of MLPs with AuNPs allowed for the visualization of the small SPIONs located inside the bilayer (Figure 8a). The inset in Figure 8a highlights the presence of small SPIONs in the hydrophobic lipidic layer, as well as of plasmonic AuNPs on the outer liposomal surface. The presence of the major building blocks employed for the synthesis of this new type of hybrid materials (liposomes, SPIONs, and plasmonic AuNPs) was also confirmed using energy dispersive spectroscopy (EDS) analysis (Figure S5).

In order to evaluate the nature of the interactions between the PEGylated AuNPs and the cationic liposomes, SERS measurements were performed in liquid solutions containing MMPNHs.

**Figure 9:**
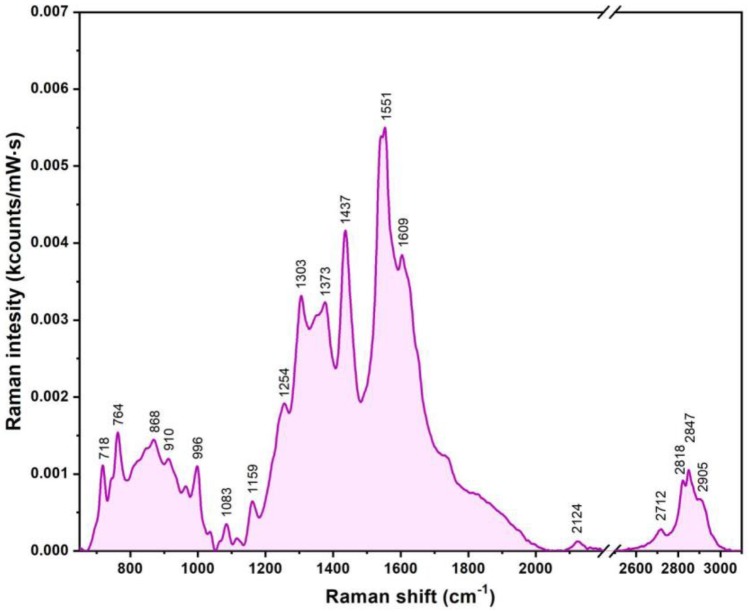
A typical SER spectrum of the fully decorated plasmonic magneto-liposomes recorded in liquid conditions using a 785 nm excitation.

In this case, the plasmonic nanoparticles decorating the liposomes acted as SERS substrates. A typical SER spectrum recorded of a liquid solution containing MMPNHs is presented in Figure 9. The major vibrational peaks, which were observed and discussed in the case of pure cationic liposomes, were also present with slightly different intensities and wavenumbers. In the low wavenumber region, the presence of the two peaks associated with choline and phosphate groups (718 and 764 cm^−1^) confirmed the fact that the electrostatic interaction of plasmonic PEGylated AuNPs with the liposomes occurred through these groups situated on the outer liposomal surface. Given the fusogenic character of PEG molecules (present on the surface of plasmonic NPs), one can suppose a strong interaction between these molecules and the choline, phosphate, and ester groups belonging to the lipid molecules. More precisely, it was possible that PEG molecules intercalated between the polar heads of the lipids. Consequently, the most intense peak present in the SER spectrum in Figure 9 (1551 cm^−1^), which was not present in the spectrum of the liposomes, could be attributed to PEG molecules. The vibrational bands associated with CH_2_ and CH_3_ groups (1303, 1437, 2818, 2847, and 2905 cm^−1^) were present at slightly different wavenumbers. Interestingly, the very intense peak located ≈1650 cm^−1^, associated with the stretching vibration of the C=C bond (present only inside the bilayer), was not present. The 1728 cm^−1^ peak, assigned to C=O bond vibrations belonging to the ester groups, probably vanished as a consequence of the interaction of the PEG molecules with these groups.

## 4. Conclusions

In this paper, the synthesis of a new type of multifunctional magneto-plasmonic nanohybrid that could have a major impact in biomedical applications related to targeted drug delivery and release applications is reported for the first time. By synergistically employing and controlling hydrophobic and electrostatic interactions acting between the major building blocks, the synthesis of this new type of multifunctional nanohybrid was realized in a very reproducible manner. Each component of the nanohybrids was thoroughly characterized by means of complementary techniques, which allowed for the understanding of their properties. Once synthesized, the multifunctional magneto-plasmonic nanohybrids were characterized using the same techniques in order to evaluate the evolution of the individual properties in this complex system. The successful incorporation of superparamagnetic iron oxide nanoparticles, together with the modifications they generated in the lipid bilayer, were analyzed for the first time by means of Raman spectroscopy performed on dry samples, whereas the plasmonic properties of the hybrids were evaluated in solutions. Based on the properties here presented, we may estimate that the synthesis of this new type of multifunctional nanohybrids could represent a major breakthrough in biomedical applications.

## Supplementary Materials

The following are available online at https://www.mdpi.com/2079-4991/9/11/1623/s1, Figure S1: X-ray diffractogram acquired on SPIONs powder; Figure S2: Zero Field Cooling and Field Cooling curves acquired on Fe_3_O_4_ spherical magnetic nanoparticles; Figure S3: Chemical structures of DOTAP and of soybean phosphatidylcholine (SPC) lipids; Figure S4: Optical image of 5 batches of liposomal dispersions ordered from left to right with respect to the volume of SPIONs dispersion used in the synthesis: 1. 0 μL (bare liposomes) 2. 10 μL 3. 50 μL 4. 250 μL 5. 1000 μL; Figure S5: Energy Dispersive Spectroscopy (EDS) analysis of MLP250 sample; Table S1: Zeta potential of nanoobjects; Table S2: PCS measurements results for the liposomal dispersions; Table S3: Assignment of the major vibrational bands recorded on cationic liposomes; Table S4. Hyperthermia properties of PEGylated AuNPs and their liposomal complexes.
