# Supplementary material for: Synergistical Use of Electrostatic and Hydrophobic Interactions for the Synthesis of a New Class of Multifunctional Nanohybrids: Plasmonic Magneto-Liposomes
Source: Nanomaterials (Basel). 2019 Nov 15;9(11):1623. doi: 10.3390/nano9111623 (PMC6915406; doi:10.3390/nano9111623)
Supplement: Supplementary file 1 [file nanomaterials-09-01623-s001.pdf]

nanomaterials

MDPI

Supplementary Materials

Synergistical Use of Electrostatic and Hydrophobic Interactions for the Synthesis of a New Class of Multifunctional Nanohybrids: Plasmonic Magneto-Liposomes

Gabriela Fabiola Ştiufiuc [1], Ştefan Niţică [2], Valentin Toma [2], Cristian Iacoviţă [3], Dietrich Zahn [4], Romulus Tetean [1], Emil Burzo [1], Constantin Mihai Lucaciu [3] and Rareş Ionuţ Ştiufiuc [2,3,*]

[1] Faculty of Physics, "Babeş-Bolyai" University, M. Kogălniceanu 1, 400084, Cluj-Napoca, Romania; gabi.stiufiuc@phys.ubbcluj.ro (G.Ş.); romulus.tetean@phys.ubbcluj.ro (R.T.); emil.burzo@phys.ubbcluj.ro (E.B.)

[2] MedFuture Research Center for Advance Medicine, "Iuliu Haţieganu" University of Medicine and Pharmacy, L. Pasteur 4-6, 400349, Cluj-Napoca, Romania; stefan_nitica@yahoo.com (Ş.N.); valentin.toma@umfcluj.ro (V.T.)

[3] Faculty of Pharmacy, "Iuliu Haţieganu" University of Medicine and Pharmacy, L. Pasteur 4-6, 400349, Cluj-Napoca, Romania; cristian.iacovita@umfcluj.ro (C.I.); clucaciu@umfcluj.ro (C.L.)

[4] Semiconductor Physics, Chemnitz University of Technology, D-09107 Chemnitz, Germany; zahn@physik.tu-chemnitz.de (D.Z.)

* Correspondence: rares.stiufiuc@umfcluj.ro

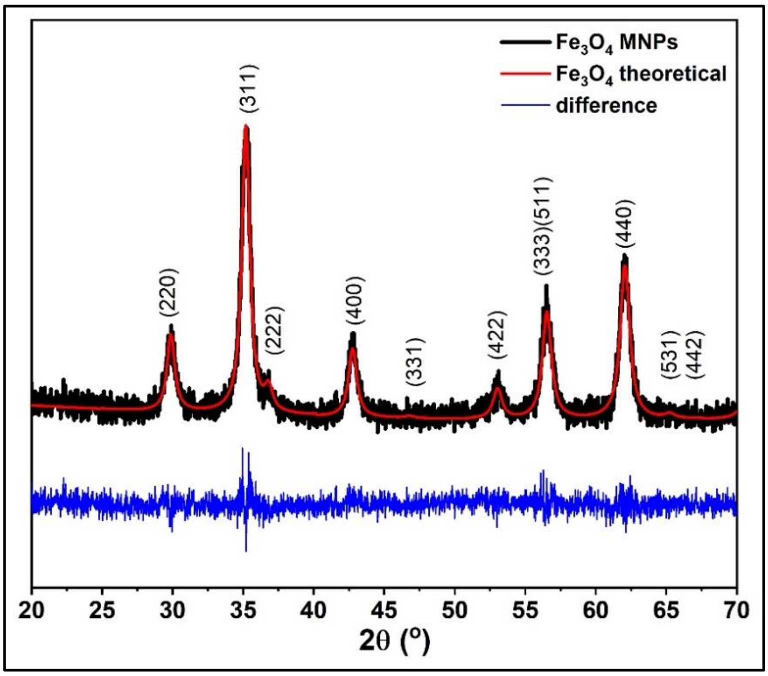

Figure S1. X-ray diffractogram acquired on SPIONs powder.

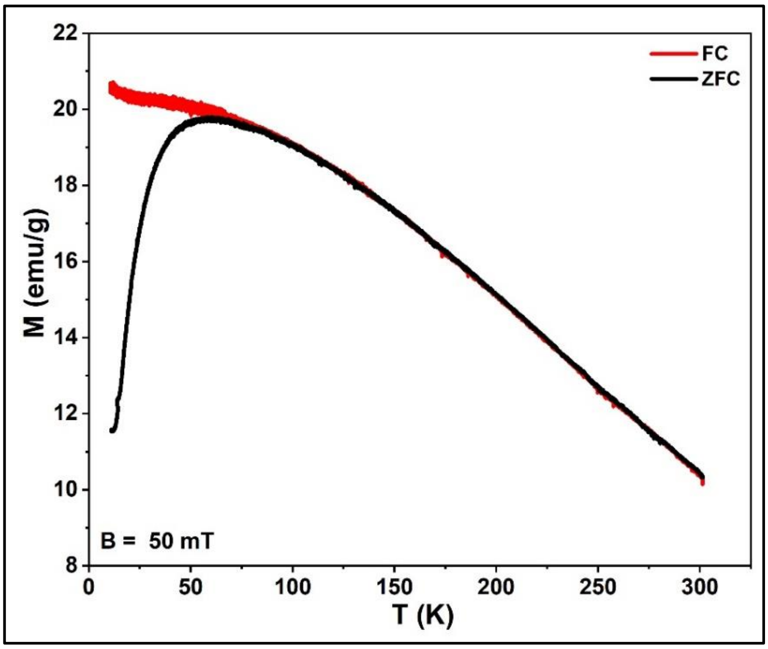

Figure S2. Zero Field Cooling and Field Cooling curves acquired on Fe_3O_4 spherical magnetic nanoparticles.

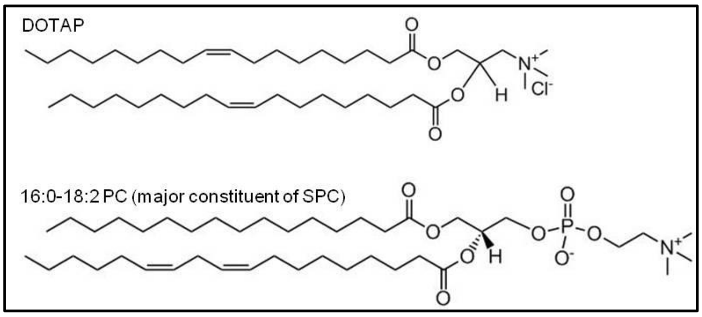

Figure S3. Chemical structures of DOTAP and of soybean phosphatidylcholine (SPC) lipids.

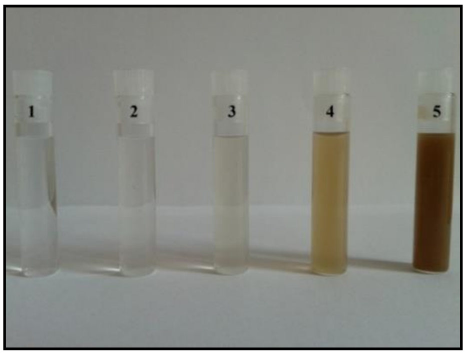

Figure S4. Optical image of 5 batches of liposomal dispersions ordered from left to right with respect to the volume of SPIONs dispersion used in the synthesis: 1. 0 μL (bare liposomes) 2. 10 μL 3. 50 μL 4. 250 μL 5. 1000 μL.

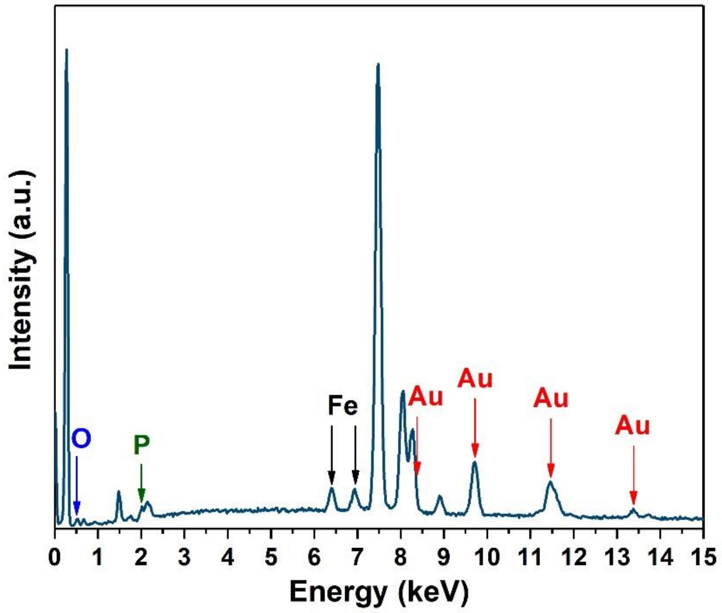

Figure S5. Energy Dispersive Spectroscopy (EDS) analysis of MLP250 sample.

Table S1. Zeta potential of nanoobjects.

Sample	Dilution	pH	ζ (mV)
AuPEG	1:16	10.1	-29.6
Lipo 0 μL	1:4	3.56	+59.7
Lipo 10 μL	1:4	3.45	+43.9
Lipo 50 μL	1:4	3.53	+43.4
Lipo 250 μL	1:4	3.99	+47.9
Lipo1000 μL	1:4	3.24	+51.1

Table S2. PCS measurements results for the liposomal dispersions.

Liposome batch	Dilution	PDI	Hydrodynamic diameter (nm)	
			Peak$_1$	Peak$_2$
Simple (0 μL)	-	0.295	180.9	31.27
10 μL	-	0.431	179.8	36.31
50 μL	-	0.342	227.5	33.55
250 μL	1:4	0.460	393.9	71.60
1000 μL	1:16	0.302	389.2	93.35

PDI = polydispersity index

Table S3. Assignment of the major vibrational bands recorded on cationic liposomes.

**Wavenumber (cm^{-1})**	**Assignment**
718	symmetric stretch vibration of the C-N bonds from the $N^{+}(CH_3)_3$ choline group
765	stretching vibration of the quaternary ammonium group & stretching vibration of the esterified P-O bonds from the phosphate groups
850	Asymmetric stretching vibration of the esterified O-P-O bonds from the phosphate groups
875	asymmetric stretch vibration of the C-N bonds from the $N^{+}(CH_3)_3$ choline group
972	bending vibration of the CH groups
1044	symmetric stretching vibration of PO_4^{3-} group
1085	stretching vibration of C-C bond
1265	deformation vibration of the unsaturated =CH group
1302	twisting vibration of CH_2 groups
1444	CH_2/CH_3 scissoring vibrations
1658	C=C bond stretching vibrations
1741	stretching vibration of the C=O bond from the ester groups
2728	in-plane scissoring vibrations of CH_2
2854	symmetric stretching vibration of the CH_2 groups (fatty acids)
2895	asymmetric stretching vibration of the CH_2 groups (fatty acids)
2929	symmetric stretching vibration of the CH_3 groups
3010	stretching vibrations of unsaturated =C-H groups (fatty acids)

Table S4. Hyperthermia properties of PEGylated AuNPs and their liposomal complexes.

Table S4. Hyperthermia properties of PEGylated AuNPs and their liposomal complexes

**Sample**	**m_{Au} (g)**	**ΔT (K)**	**SAR (W/g)**
PEGylated AuNPs	$4.115\cdot10^{-5}$	2.61	327
MMPNHs	$4.113\cdot10^{-5}$	3.08	450
